# Immune Checkpoints in Endometriosis—A New Insight in the Pathogenesis

**DOI:** 10.3390/ijms25116266

**Published:** 2024-06-06

**Authors:** Dorota Suszczyk, Wiktoria Skiba, Anna Pawłowska-Łachut, Izabela Dymanowska-Dyjak, Karolina Włodarczyk, Roman Paduch, Iwona Wertel

**Affiliations:** 1 Independent Laboratory of Cancer Diagnostics and Immunology, Medical University of Lublin, Chodźki 1, 20-093 Lublin, Poland; dorotasuszczyk@umlub.pl (D.S.); wiktoria.skiba@umlub.pl (W.S.); anna.pawlowska@umlub.pl (A.P.-Ł.); karolina.wlodarczyk@umlub.pl (K.W.); 2 Independent Laboratory of Minimally Invasive Gynecology and Gynecological Endocrinology, Medical University of Lublin, Staszica 16, 20-081 Lublin, Poland; i.dymanowska@gmail.com; 3 Department of Virology and Immunology, Institute of Microbiology and Biotechnology, Faculty of Biology and Biotechnology, Maria Curie-Skłodowska University, Akademicka 19, 20-033 Lublin, Poland; roman.paduch@mail.umcs.pl

**Keywords:** endometriosis, immune checkpoints, immunosuppression, immune cells

## Abstract

Endometriosis (EMS) is an oestrogen-dependent, chronic disease affecting women of a reproductive age. One of the important factors involved in the development of this disease is the complex disorders associated with the functioning of the immune system. Recent evidence has shown that EMS development is associated with changes in systemic and local immunity, including functional disturbances of effector and antigen-presenting cells. One of the reasons for immune imbalance can be the improper expression of immune checkpoints (ICPs). ICPs and their ligands are responsible for maintaining self-tolerance and the modulation of the initiation, duration, and magnitude of the immune response of effector cells in normal tissues to avoid tissue damage. Considering the complex nature of co-stimulatory or co-inhibitory ICPs and the signalling between effector cells and APCs, we hypothesise that changes in cells’ activity caused by ICPs may lead to serious immune system disturbances in patients with endometriosis. Moreover, both upregulation and downregulation in the expression of ICPs may be implicated in this process, including the reduced activity of effector cells against endometrial implants and disturbances in the antigen-presenting process. In this narrative review, we discuss, for the first time, key findings from the emerging literature, describing the associations between ICPs and their possible implication in the pathogenesis of endometriosis.

## 1. Introduction

Endometriosis (EMS) is a chronic inflammatory gynaecological disease that is defined as the abnormal growth of endometrial tissue outside of the uterus. EMS has a strong negative impact on women’s quality of life due to painful menstrual periods, chronic pelvic pain, dyspareunia, heavy menstruation, and painful defecation/urination. Endometriosis is considered one of the most important causes of infertility. It is estimated that EMS is diagnosed in about 50% of women who struggle with getting pregnant [1]. The presence of EMS is connected with mental health disorders which result in the lower overall wellbeing of patients, such as anxiety, depressive symptoms/depression, and also relationship problems [2].

It is worth highlighting that the incidence and prevalence of endometriosis are difficult to quantify, considering that some patients with this pathology are asymptomatic. For many years, the laparoscopic method was necessary to confirm endometriosis, which makes diagnosis more difficult. Even though considerable progress has been achieved in the field of the pathogenesis of endometriosis in recent years, its treatment is still challenging. Currently, the efficacy of the standard of care has reduced, and there is no successful medical or surgical treatment for this disease. It is also worth noticing that the clinical presentation of EMS, treatment response, or prognosis may not frequently correlate with its classification and staging. It is now clear that the aetiology of endometriosis is multifactorial and involves anatomical, hormonal, immune, genetic, epigenetic and environmental components. Moreover, their interactions are complex, and, predictably, the disturbance in one contributes significantly to several alterations in the other [3,4].

Recently, new evidence has started to shed light on co-inhibitory immune checkpoints that might be related to endometriosis pathophysiology and that, so far, were outside our focus. In this narrative review, we present the role of the immunological aspects of endometriosis development, focusing on immune checkpoints (ICPs) which may be implicated in the development and progression of endometriosis.

## 2. Methodology

To prepare this narrative study, a comprehensive literature overview was conducted using the PubMed/Medline/Scopus database. The search strategy process included a combination of medical subject headings (MeSH) terms and keywords, including “endometriosis”; “immune checkpoint”; “ICPs”, “immune checkpoint in endometriosis”; “co-inhibitory checkpoints”; “programmed death receptor 1”; “PD-1”; “programmed death ligand 1”; “PD-L1”; “programmed death ligand 2”; “PD-L2”; “T-cell immunoglobulin and mucin domain 3”; “TIM-3”; “Galectin-9”; “Gal-9”; “cytotoxic T cell antigen 4”; “CTLA-4”; “T cell immunoreceptor with Ig and ITIM domains”; “TIGIT”; “DNAM”; “CD226”; “CD155”; “CD200”; and “CD200R”. Concerning the literature screening methodology and study selection criteria employed, only English full-length articles published in international peer-reviewed journals were included. The study selection process was restricted to human subjects. Finally, relevant data were retrieved via manual citation mining.

## 3. The Immune System in Patients with Endometriosis

Despite numerous theories trying to understand the pathogenesis of EMS, none of them have determined the exact mechanism of the disease development. The oldest retrograde menstruation theory was presented by Sampson in 1927. He assumed that the presence of retrograde menstrual efflux with little fragments of endometrium passed through the fallopian tubes during menstruation to the peritoneal cavity, giving rise to ectopic endometrial implants. However, this theory turned out to be insufficient to clarify the exact process of EMS development, largely because retrograde menstruation is a physiological process that occurs in 90% of women. What is unusual in patients suffering from endometriosis that only 10% of them develop the disease? The possible answer to this question was discovered by Dmowski et al. They suggest that in patients with EMS, a defective immune response occurs that prevents the elimination of the menstrual debris and promotes the implantation and growth of endometrial cells in the ectopic sites. This state may be connected with immunodeficiency due to the immunocompromisation of the immune system’s ability to remove implants [5,6].

Over the years, researchers have discovered significant quantitative and functional changes in the proper activity of neutrophils, monocytes/macrophages (Mo/Ma), natural killer cells (NKs), dendritic cells (DCs), and B or T cells on the periphery and in the peritoneal cavity in women suffering from endometriosis. Moreover, in these patients, the presence of immunosuppressive cells like myeloid-derived suppressor cells (MDSCs), regulatory T cells (Tregs), and regulatory B cells (Bregs) have been observed, which not only suppress a proper immune response, but also promote the disease progression [7,8,9]. Scheerer et al. described an elevated infiltration of immune cells (T and B cells, macrophages) in the implants and eutopic endometrial tissue of EMS patients. They called this phenomenon endometriosis-associated immune cell infiltrates (EMaICI). Interestingly, they also demonstrated that the T cell CD3^+^, CD4^+^, and CD8^+^ phenotypes had larger sizes, especially in peritoneal and ovarian EMS, but they are ineffective in clearing endometrial cells [10].

The growth of the ectopic endometrial implants leads not only to the recruitment of immune cells, but also to intense chronic inflammatory responses with elevated levels of cytokines, chemokines, and growth factors like IL-6, IL-1, IL-4, IL-25, IL-8, IL-10, IL-37, GM-CSF, VEGF, HGF, TNF-α, and TGF-β being implicated in the establishment and persistence of endometrial lesions [11,12]

Accumulating evidence showed that the inflammatory milieu plays an essential role in both EMS and ovarian cancer pathogenesis. The development of inflammatory conditions may cause a defective “immune surveillance” that prevents the elimination of implants and promotes their implantation and growth in the ectopic sites outside of the uterus [13]. Intriguingly, it seems that the development of endometriosis closely resembles cancer. EMS implants exhibit the ability to proliferate, angiogenesis, the invasion of nerve fibres, and the ability to avoid apoptosis, leading to the infiltration of the surrounding tissue [14,15]. Various investigations have provided data supporting the observation that endometriosis may transform into endometriosis-associated ovarian cancer (EAOC)—clear-cell, endometrioid, and low-grade serous ovarian cancers. EAOC development is connected with the presence of multifaceted factors like endocrine imbalance, the presence of the oxidative stress, and disturbances in the immune system with chronic inflammation, which are also described in endometriosis [16].

It is worth highlighting that women with endometriosis develop immunosuppression, but the exact mechanisms are poorly understood. Researchers supposed that it is similar to the mechanisms present in cancers, including the reduced antigen-presenting capacity, the increased secretion of immunosuppressive cytokines (IL-10, TGF-β), the decreased expression of major histocompatibility complex (MHC) antigens on the surface of antigen-presenting cells (APCs), and the accumulation of cells with immunosuppressive potential, e.g., MDSCs, Tregs, tumour-associated macrophages (TAMs) [17,18,19]. However, one of the most common mechanisms implicated in tumour escaping and the suppression of the immune system response against cancer cells is an elevated expression of inhibitory immune checkpoints. It results in suppressing the effector functions of T cells and NK cells [20].

We hypothesise that disturbances in the proper activity of effector cells and APCs, the presence of “exhausted” T cells, and immunosuppression in patients suffering from endometriosis are the result of an immune imbalance in the expression of immune checkpoints.

## 4. Immune Checkpoint Molecules

Over the years, numerous studies focused on the role of co-inhibitory and co-stimulatory molecules in regulating the T cell activation and homeostasis of the immune system, known as immune checkpoints. ICPs and their ligands are key players in maintaining self-tolerance and the modulation of the initiation, duration, and magnitude of the immune response of effector cells in normal tissues to avoid tissue damage [19].

Based on their roles in regulating immunity, ICPs can be divided into co-inhibitory molecules, such as programmed cell death receptor-1 (PD-1), lymphocyte activation-gene-3 (LAG-3), T-cell membrane protein-3 (TIM-3), and cytotoxic T-lymphocyte antigen-4 (CTLA-4), and co-stimulatory molecules, such as CD28, TNF receptor superfamily member 9 (4-1BB), TNF receptor superfamily member 4 (OX40), inducible co-stimulatory molecule (ICOS), or glucocorticoid-induced TNF receptor family-related protein (GITR) and their ligands [21,22].

In numerous cancers, the increased expression of ICPs and their ligands is a known mechanism of tumour cell escape from the control of the immune system. The elevated expression of inhibitory ICPs is associated with reduced T cells’ effector function, leading to their apoptosis or ‘exhaustion’. It is clear that exhausted T cells exhibit defective proliferative capacity, and their effector functions, such as cytotoxicity and cytokine production, are impaired. In contrast to inhibitory receptors, co-stimulatory ICPs are responsible for T-cell activation via providing the co-stimulatory second signal during the immune response [19].

Considering the complex nature of co-stimulatory or co-inhibitory signalling between effector cells and APCs, we hypothesise that an imbalance in their activity can lead to serious immune system disturbances in patients with endometriosis. Moreover, both upregulation and downregulation in the expression of ICPs is implicated in this process, including the reduced activity of effector cells against endometrial implants.

ICPs can be involved in both the early steps of the initiation of endometriosis and also in its progression via the development of tolerance on endometrial implants and immunosuppression on the periphery and in the peritoneal cavity, favouring their growth and proliferation.

## 5. The PD-1/PD-L1/PD-L2 Pathway in Endometriosis

In recent years, the PD-1/PD-L1/PD-L2 pathway has been one of the most studied immune checkpoints responsible for the negative regulation of T-cell activity. The expression of the PD-1 receptor was described on activated T cells, B cells, NK cells, Mo, and DCs. The ligand PD-L1 (less PD-L2) is expressed on DCs, Mo/Ma, and numerous cancer cells, including melanoma, ovarian, breast, cervical, hematologic cancers, and non-small cell lung cancer. An immune checkpoint can also occur in soluble forms like sPD-1 or sPD-L1/sPD-L2 [23]. The mechanism of this pathway is based on the interaction between ligands PD-L1 or PD-L2 and receptor PD-1, which leads to the deactivation of T cells through hampering CD28 signalling. The loss of proliferation activity and the decreased production of cytokines (like IL-2, TNF-α, and IFN-γ) result in T cell exhaustion and apoptosis. Findings from numerous studies have shown that cancer cells suppress an antitumor immune response through expressing PD-L1 on their surface or inducing the expression of PD-L1 on tumour-associated immune cells like Ma or DCs [24]. It has been reported that PD-1 expression can be found on cancer cells in melanoma, liver, renal, testis, and urothelial cancer. In these malignancies, we observed subgroups of tumour cells with positive PD-1 staining, detected with immunochemistry [25]. The expression of PD-L1 was described in melanoma, ovarian, breast, cervical, hematologic cancers, and non-small cell lung cancer [23]. On the other hand, PD-L2 expression was observed in melanoma, non–small cell lung cancer, head and neck squamous cell carcinoma, triple-negative breast cancer, and renal, bladder, or gastric cancer, and can also occur in the absence of PD-L1 [26].

Recent evidence suggests that the PD-1/PD-L1 pathway is supposed to be particularly relevant in the pathogenesis of endometriosis. Nevertheless, in the literature, there are limited studies on the expression of these ICPs in patients with EMS.

Firstly, Walankiewicz et al. examined the expression of receptor PD-1 and its ligand PD-L1 on the surface of T and B cells in the peripheral blood of both patients with EMS and healthy women. According to their study, CD4^+^ CD8^+^ T cells and CD19^+^ B cells with PD-1 and PD-L1 expression were found more frequently in women with endometriosis in both early (I/II) and late (III/IV) endometriosis stages, according to the American Society for Reproductive Medicine (ASRM), as compared to healthy individuals. Additionally, patients with adhesions had lower frequencies of CD8^+^PD-L1^+^ T cells than those determined in the other EMS groups. It is important to note that the study had a small sample size, consisting of only twenty-five patients divided into two groups (I/II *n* = 18 and III/IV *n* = 7), and did not measure the activity of T cells in EMS patients. The authors suggest that disturbances of the PD-1/PD-L1 axis may be related to the continuous activation of T cells, the development of autoimmunity, and the maintenance of an inflammatory process in these patients [27]. In our previous study, we focused attention on the expression of PD-L1 and PD-L2 ligands on myeloid and plasmacytoid DCs in the peripheral blood (PB) and peritoneal fluid (PF) of women with EMS. We observed that the percentages of both mDCs and pDCs with PD-L1 and PD-L2 expression were significantly higher in the PF than detected in the peripheral blood of EMS patients. Moreover, we compared the percentage of mDCs and pDCs with PD-L1 or PD-L2 expression in the PB of EMS patients with the percentage of these cells in healthy women. We detected significantly lower percentages of PD-L1 positive mDCs/pDCs and PD-L2 positive mDCs in the peripheral blood of the EMS group in comparison to the control group. The percentage of PD-L2 positive pDCs was lower in the PB of EMS patients in comparison to the control group. Based on the previous studies concerning the role of ICPs in other diseases like cancer, we concluded that changes in PD-L1/PD-L2 expression on the surface of mDCs and pDCs in patients with EMS may lead to disturbances or even the inhibition of the proper T cell activation, and the development of immunosuppression, supporting the implantation, proliferation, and growth of endometrial tissue in the peritoneal cavity [28].

In 2019, Wu et al. showed that eutopic and ectopic (glands and stroma) endometrial tissues in patients suffering from EMS exhibited a higher level of PD-1 expression. In contrast, healthy tissues exhibited very weak immunostaining for PD-1. Similarly, PD-1 was expressed at higher levels in the ectopic endometria of EMS tissues than in normal or eutopic endometria in EMS according to Western blot analysis. It was confirmed that PD-L1 is not expressed or only weakly expressed in normal tissues. In contrast, according to the immunohistochemical and Western blot analysis, it was expressed at significantly higher levels in both eutopic and ectopic EMS tissues. In addition, the expression of PD-L1 was higher in the ectopic endometria than in the eutopic endometria of the EMS group. Using flow cytometry, the authors also reported the upregulated expression of PD-1 and PD-L1 in CD4^+^ and CD8^+^ T cells from the EMS patients in comparison to the control group. They concluded that changes in the immune system activity caused by the elevated expression of PD-1 and PD-L1 on T cells may be an important factor in the early steps of EMS development, which is similar to the mechanism observed in cancer (e.g., ovarian cancer), where tumour cells can evade the immune response via the upregulation of the PD-1/PD-L1/PD-L2 axis [29].

Recently, Okşaşoğlu demonstrated that PD-1 and PD-L1 levels, determined by the enzyme-linked immunosorbent assay (ELISA), were increased in the serum of patients with EMS when compared to healthy subjects. Interestingly, the level of PD-1 was approximately eight-fold higher and the level of PD-L1 was approximately four-fold higher than that in the control group. Moreover, using qRT-PCR, they found higher expressions of PD-1 in the EMS group than in the control [30]. Similarly, Santoso et al. showed elevated serum levels of sPD-L1 in EMS patients (especially in the late stages of EMS) when compared to the control group [31]. On the contrary to Okşaşoğlu, in their study, there was no difference in the sPD-1 level between the EMS and control group. sPD-1 was higher, especially in the late stages of EMS, whereas sPD-L1 was higher in both the early and late stages of EMS when compared to the control group. It is worth noting that their control group consisted of women with a single benign gynaecologic disorder related to the fallopian tubes, ovaries, or fibroids, and not healthy subjects. Moreover, the level of sPD-L1 in the serum was nearly twice as high in the EMS-related infertility group in comparison to the control group [31].

In our previous study, the concentration of sPD-L1 was significantly lower in the plasma of EMS patients than that detected in the healthy subjects. Interestingly, we showed a higher accumulation of sPD-L1 in the peritoneal fluid than in the plasma of EMS patients. For the first time in our study, we determined the level of PD-L2, which was elevated in PF than in the plasma of EMS patients. The soluble form of PD-L1 or PD-L2 may play an important role in the modulation of the immune response and the development of tolerance on implants not only in the peritoneal cavity, but also in the periphery, supporting implantation endometrial tissue in the distance from the uterine cavity [28]. Hosseinzadeh et al. in 2024 examined the expression of PD-1 on the surface of NKs and NKT cells using flow cytometry. They found a higher percentage of PD-1-positive NKs in the peritoneal fluid of patients with EMS than in the control group. They did not find differences between the percentages of NKT with PD-1 expression in the PF and PB of patients with and without EMS. An elevated expression of the PD-1 receptor on NK cells in patients with EMS may be responsible for their dysfunctional and even exhausted state, which is similar to “exhausted T” cells. Such NKs have decreased effector/cytolytic activity and are ineffective in the removal of endometrial implants from the peritoneal cavity [32].

EMS is considered an oestrogen-dependent disease and oestrogen may be engaged not only in the development of inflammation, but also in angiogenesis, promoting the proliferation of endometrial cells in patients with EMS [33]. Wu et al. showed that treatment with oestrogen E2 increased the expression of PD-L1 in epithelial cells isolated from the eutopic endometrium of patients suffering from EMS. Interestingly, the increase in PD-L1 expression was independent of the dose of E2. On the contrary, they documented that PD-L1 expression in endometrial cells is not regulated by IFN-γ or TNF-α [29].

It was proven that the presence of PD-L1 on tumour cells and APCs leads to the induction of T cell apoptosis, their anergy or exhaustion, elevated IL-10 production, abnormalities in the proper function of DCs, and the elevated differentiation of Tregs. It helps cancer cells escape from the control of the immune system and protects them from lysis with cytotoxic T lymphocytes. It is commonly known that the expression of PD-L1 is weak in most normal tissues, whereas it may grow in response to cancer-associated inflammation. However, cancer cells also express PD-L1 in IFN-γ-independent manners, similar to constitutive signalling by anaplastic lymphoma kinase (ALK), *EGFR* mutations, and the loss of phosphatase and tensin homologs (*PTEN*) [34]. Similar mechanisms of immunosuppression may occur in patients with endometriosis, supporting endometrial implants in evading the host immune system response. Genetic mutations in *ARID1A*, *KRAS*, *PTEN*, *PIK3CA*, and the loss of heterozygosity were found in both endometriosis and EAOC, and may be engaged in the progression of EMS into ovarian cancer [35]. It is worth highlighting that the loss of the *PTEN* gene has an impact on modifying cytokine secretion and supports creating an immunosuppressive microenvironment [36]. The inactivation of *PTEN* was identified as an early step in the development of malignant transformation EMS localized on the ovary [37]. Nero et al., in their retrospective case–control study, showed that decreasing infiltrating T lymphocytes (ITLs) and increasing PD-1/PD-L1 may be one of the most important factors in transforming the EMS into EAOC [16]. Additionally, one-third of EMS cases (localized on the ovary) occur in the EAOC-like PD-1/PD-L1 expression profile. They also observed no differences between EAOC and atypical endometriosis samples from the same patients supporting the hypothesis that the presence of atypical endometriosis may be a precursor of EAOC. These observations suggest that changes in an immune system supporting the development of cancer cells occur earlier, which are believed to be benign conditions [16].

Considering the presented data, it seems that the PD-1/PD-L1/PD-L2 axis plays a role not only in endometriosis development, but also in the progression of EMS into EAOC and EMS-related infertility. Moreover, this pathway regulates immunological tolerance on endometrial implants in patients with EMS through higher PD-1 and PD-L1 expression levels in eutopic and ectopic endometrial tissues and the elevated expression of PD-1 and PD-L1 on immune cells like T or B cells. Additionally, an elevated percentage of PD-L1/PD-L2-positive mDCs and pDCs and higher levels of the soluble form of PD-L1 and PD-L2, especially in the PF, may hamper the proper immune response in these patients, decreasing the antigen capacity of DCs, and leading to the development of local immunotolerance and even immunosuppression in the peritoneal cavity.

## 6. The TIM-3/Gal-9 Pathway in Endometriosis

The second important axis that negatively regulates T cell activation and function is receptor TIM-3 with its ligand galectin-9 (Gal-9). TIM-3 can be found on Th1, Th17, Mo/Ma, and DCs, whereas Gal-9 is expressed on the surface of eosinophils, T cells, Ma, or DCs [38,39]. The mechanism of activity of the presented pathway is based on the interaction between TIM-3 and Gal-9, which hamper the immune response mediated by Th1, Th17, and Tc1 cells. T cells have an impaired ability to proliferate and engage in cytokine production (IL-2, IFN-γ, and TNF-α), but this axis also leads to T cell death [39]. In healthy conditions, the TIM-3/Gal-9 pathway regulates T-cell development and homeostasis and the inflammatory response, assists the maturation of dendritic cells, and prevents autoimmune conditions and fetomaternal tolerance during normal pregnancy [40,41].

Due to its specific function in regulating the various crucial mechanisms essential for maintaining cell homeostasis, the TIM-3/Gal-9 pathway has become the focus in the pathogenesis of endometriosis. Firstly, in patients with EMS, Brubel et al. found an overexpression of Gal-9 mRNA in the eutopic endometrium in comparison to the Gal-9 mRNA level detected in the control group. Moreover, ectopic implants expressed higher elevated Gal-9 mRNA levels than in the control tissue drawn from the surrounding area. The authors also showed that Gal-9 expression was elevated in cells from PF in the EMS group. Their results suggest that not only eutopic endometrium or ectopic implants but also immune cells from the peritoneal fluid may be the source of Gal-9 in patients suffering from EMS [42]. Also, Tian et al. described significantly higher levels of TIM-3 mRNA and protein expression in ectopic and eutopic endometrial tissues in patients with EMS than in normal endometrium. In addition, they discovered that TIM-3 may have an impact on endometrial stromal cell (ESC) proliferation and endometriotic lesion development via the brain-derived neurotrophic factor (BDNF)-mediated PI3K/AKT pathway [43].

Meggyes et al. documented the significantly lower expression of TIM-3 on the surface of CD3^+^, CD8^+^, and CD4^+^ T cells and significantly elevated levels of TIM-3 on the FoxP3^+^ Tregs in the PB in patients with EMS compared to those without. They described a decreased expression of TIM-3 on the surface of all CD56^+^ NK cell subsets in the PB from patients suffering from endometriosis. On the contrary, all examined subsets of T cells from PF showed increased TIM-3 expression in comparison to T cells from PB. In addition, on NKT-like cells from peritoneal fluid in patients with EMS, they detected an elevated expression of TIM-3 [44]. The authors continued experiments under Gal-9 expression in patients with endometriosis, and, using flow cytometry analysis, demonstrated the higher expression of Gal-9 on CD4^+^ T and Tregs cells in the PB of patients with EMS in comparison to healthy women. Additionally, Gal-9 expression was increased on the surface of CD3^+^ T, CD8^+^ T, and Treg cells in the PF when compared with PB in the EMS group. They also described significantly increased Gal-9 expression on the total NK, the CD56^bright^ NK, and CD56^dim^ NK cells in the PF than in the PB of the EMS group. Meggyes et al. suggest that changes in TIM-3 and Gal-9 expression may alter the proper function of immune cells, supporting the survival of ectopic implants [44]. Also, Hosseinzadeh et al. examined the TIM-3 expression on the surface of NKs and NKT cells using flow cytometry, but they found no difference in the frequency of both TIM-3^+^ NKs and TIM-3^+^ NKT cells between EMS and control groups in both PF and PB [32].

In our previous study, we evaluated the expression of Gal-9 on antigen-presenting cells and observed a higher accumulation of myeloid and plasmacytoid DCs Gal-9^+^ in the peritoneal fluid than in the peripheral blood of EMS patients. Moreover, the percentage of mDCs Gal-9^+^ was significantly higher in the late (III/IV) ASRM stages of EMS than those determined in the PB of the control group [45]. In addition, we observed the accumulation of sGal-9 and sTIM-3 in the PF of the EMS patients. The sGal-9 level was higher in the plasma of both the early (I/II) and late (III/IV) ASRM stages of EMS than that detected in the control group, whereas the level of sTIM-3 was higher in the plasma in the late stages (III/IV) of EMS [45]. In line with our results, Brubel et al. showed an elevated level of sGal-9 in the serum of EMS patients in comparison to healthy women [42]. Interestingly, Jarollahi et al. described that the level of sGal-9 measured in the serum of EMS patients represents high sensitivity (100%) and sufficient specificity (88.46%), allowing this to be a helpful diagnostic tool and to be compared to the laparoscopy method [46].

The current findings indicate that receptor TIM-3 is highly expressed on many types of tumour cells like liver, colon, prostate, cervical, or ovarian cancer, promoting invasion, migration, and tumour progression. Many studies have addressed the role of the TIM-3/Gal-9 pathway in driving T-cell exhaustion in tumours and chronic infections. This axis also causes peripheral immune tolerance [38,39]. Cancer cells use ICPs to escape from the anti-tumour immune attack. Gal-9 is engaged in immune escaping through promoting the differentiation of Tregs, and inducing the apoptosis of Th and Tc cells. Moreover, it has been observed that Gal-9 is responsible for angiogenesis, which is essential for tumour progression [47]. The TIM-3/Gal-9 pathway has an impact on the activity of DCs and suppresses their function, inhibiting anti-tumour immunity and promoting inflammatory conditions [39].

It seems possible that elevated expression of Gal-9 on DCs can successfully hamper their proper activity to present antigens in patients with EMS. Moreover, Gal-9 could be responsible for the development of the local inflammation, leading to the infiltration of NK cells, neutrophils, T cells, Ma, and DCs into the site of implants, especially in the early stages of EMS. An increased level of Gal-9 may be connected with the immune imbalance, the dominance of the Th2 immune response, the enhancer differentiation of suppressive Tregs cells, and the progression of the disease [39].

Previous data indicate that the expression of TIM-3 can be a marker for mature and functional NK cells, and the high expression of this receptor displays the most potent ability for cytokine production and the cytotoxicity of NK cells. Interestingly, in patients with chronic HIV-1 infection connected with immunodeficiency, it was discovered that constant exposure to soluble Gal-9 leads to NK cell degranulation and TIM-3 downmodulation [48]. In patients with endometriosis, similar constant exposure to the soluble form of Gal-9 can lead to lower expressions of TIM-3 on NK cells.

## 7. The CTLA-4/CD80/CD86 Pathway in Endometriosis

Another important inhibitory ICP in the maintenance of immune homeostasis is CTLA-4, which belongs to the family of type I membrane receptors. CTLA-4 can be found on the surface of T cells after antigen stimulation, but it is also constitutively expressed on Tregs. CTLA-4 is responsible for decreasing T-cell activation and favouring their anergic state with a lower ability to proliferate and inhibit IL-2 production. Ligands for CTLA-4, CD80 and CD86, can be found on the surface of APCs and can interact not only with CTLA-4, but also with CD28, providing positive or negative stimulation for T cells. The inhibitory mechanism of CTLA-4 is based on preventing the continuous activation of the T cells, which may lead to an excessive response of the immune system, damaging tissues. On the other hand, the expression of CTLA-4 on the surface of Tregs is an important factor in avoiding autoimmunity and regulating the proper function of APCs and naïve T cells. It has been proven that the presented ICPs are essential in cancer development and represent an attractive target in immunotherapy [49,50].

Interestingly, previous evidence demonstrates that CTLA-4 may be implicated in the pathogenesis of endometriosis, but the exact mechanisms are still unclear, and the data are conflicting. Considering the autoimmune basis of EMS development, Viganó et al. in 2005 decided to examine the CT60A/G dimorphism and +49A/G polymorphism of the CTLA-4 gene, which may be related to EMS. Still, they did not observe statistically significant differences in the presented genotypes between patients suffering from EMS and women without EMS. They summarized that endometriosis development is not primarily connected with CTLA4-linked autoimmunity [51]. In line with the results presented by Vigano et al., Lerner et al. did not find a correlation between polymorphisms of CTLA-4 +49A/G and infertility or idiopathic infertility in EMS patients when compared to the control group [52].

Recently, Santoso et al. found a higher concentration of the soluble form of CTLA-4 (sCTLA-4) in the serum in the late stage (III/IV) of EMS patients than that determined in the early stage (I/II) and in control individuals (consisting of benign gynaecologic disorders of fallopian tubes, ovaries, or fibroids). In addition, the concentration of sCTLA-4 in the peritoneal cavity in patients with endometriosis was elevated when compared to the control group, with a significantly higher level in the advanced stages of EMS [31]. Similarly, Abramiuk et al. evaluated the concentration of soluble CTLA-4 in plasma and peritoneal fluid, and they found a higher concentration of CTLA-4 in the PB of women with EMS than in the control group consisting of healthy blood donors. However, no differences were described in the concentration of sCTLA-4 in the plasma and PF in the EMS group in correlation to the presence of pelvic pain syndrome, infertility, and adhesions [53].

They also documented an elevated percentage of CD8^+^CTLA-4^+^ T cells in the PB from the EMS group than in the control group. In this study, there was a positive correlation between the percentage of CD4^+^ and CD8^+^ T cells with CTLA-4 expression and the severity of the disease. The authors observed an elevated percentage of CTLA-4 positive T cells in EMS women with adhesions [53].

An intriguing result was presented by Gueuvoghlanian-Silva et al., who found that women with endometriosis had a significantly higher percentage of Tregs with CTLA-4 expression in the PB than with benign gynaecologic disorders without endometrial implants in the peritoneal cavity. Additionally, a higher percentage of CTLA4^+^ Tregs was observed in PF in comparison to the peripheral blood in these patients [54]. It has been proven that the presence of the constitutive expression of CTLA-4 on Tregs is one of the crucial components mediating immunosuppression during antitumor immunity [55]. Moreover, CTLA-4 positive Tregs alter or inhibit interactions between T cells and APCs, leading to an impaired immune response [56], and are involved in immunosuppression development, especially in the peritoneal cavity, via the downregulation of co-stimulatory molecules on the surface of APCs [54]. Data from clinical studies show that CTLA-4 can be implicated in the maintenance of persistent inflammation in the peritoneal cavity [57], and that an elevated concentration of sCTLA-4 in PF in patients with EMS may have a tolerogenic function, which is similar to that observed in malignancy. Interestingly, high levels of sCTLA-4 were detected in serum individuals with melanoma, acute B cell lymphoblastic leukaemia, and mesothelioma [58]. An interesting observation was that the soluble form of CTLA-4 featured strong suppressive activity on CD8^+^ T cells, and they exhibited a reduced ability to proliferate in the presence of sCTLA-4 in mice models [59]. On that basis, receptor CTLA-4 can be engaged in the pathogenesis of endometriosis, but it will take time to present the precise regulatory mechanism of the presented ICPs in EMS implant development.

## 8. Other Less Common ICPs in Endometriosis

Little is known about the other inhibitory ICP pathways like T-cell immunoglobulin and the immunoreceptor tyrosine-based inhibitory motif domain (TIGIT)/DNAM (CD226)/CD155 or CD200/CD200R in the pathogenesis of endometriosis. Only one group of researchers examined the expression of inhibitory receptors TIGIT and CD226 on peripheral CD4^+^ T cells, but they did not find any differences in the CD4^+^ TIGIT^+^ T cells between the EMS group and the control individuals. On the other hand, the expression of CD226 on CD4^+^ T cells was lower in EMS in comparison to the control group. Li et al. found that the levels of cytokines like TNF-α, IL-10, and IFN-γ were higher in TIGIT^+^ and CD226^+^ CD4^+^ T cells when compared to TIGIT^−^ and CD226^−^ CD4^+^ T cells. However, the data are preliminary and insufficient to prove the role of TIGIT and CD226 in the pathogenesis of endometriosis. This axis needs further extensive investigation [60].

The last axis with a possible impact on EMS development is CD200/CD200R, engaged also in the pathogenesis of allergies, autoimmune diseases, and cancers. CD200 can be found on cancer cells like sarcomas, melanoma, and especially in brain tumours, whereas CD200R was detected on MDSCs, Ma, and DCs, and rarely on T cells. The results of the interaction between CD200R and CD200 depend on the kind of tumour and the inflammatory milieu. However, it was confirmed that CD200 promotes immunosuppression through increasing the suppressive activity of MDSCs. MDSCs hamper the antitumor effect of NK and T cells, favouring tumour growth. Moreover, CD200/CD200R is known for hampering the response to immunotherapy through promoting the expansion and activity of strong immunosuppressive MDSCs [61,62]. It is worth mentioning that patients with EMS had an elevated percentage of MDSCs in the PB in comparison to healthy women [63,64]. It is supposed that the infiltration of MDSCs is engaged in the early stages of EMS development and is considered a critical step for the survival of EMS implants via inhibiting the proliferation/proper function of both CD4^+^ and CD8^+^ T cells, thus promoting immunosuppression and supporting angiogenesis. Moreover, MDSCs may also play an important role in the progression of the EMS into EAOC [65]. Weng et al. discovered that CD200 can decrease Ma proliferation and have an impact on their low phagocytic activity. Moreover, the upregulation of the expression of CD200 caused by oestrogen decreases the activity of Ma, resulting in their “disfunction state”. An elevated percentage of immunosuppressive MDSCs (which can weaken the activity of T or NK cells) and CD200 may be one of the key factors increasing the percentage of MDSCs in EMS patients [66].

Taking into account the presented function, Clark et al. in 2018, using immunochemistry, demonstrated for the first time that CD200 and CD200R proteins were present in EMS implants (in stroma and epithelium) [67]. Next, in 2022, Clark et al. documented that in EMS patients, CD200S^+^ stromal cells were more numerous than CD68^+^ Ma, and these cells were similar in both number and location to CD56^bright^ endometrial NKs. The frequency of CD200S^+^ cells was found to be greater in the stroma surrounding the smaller ectopic cysts. They observed that the frequency of CD200S^+^ NKs may be greater in the endometrium of patients suffering from endometriosis [68]. Hamilton et al. found elevated serum levels of CD200 during the secretory phase in women with EMS in comparison to the control group. Moreover, the level of CD200 in the serum correlated with a matched level of CD200 mRNA in the endometrium. On the other hand, they observed a correlation between a lower level of circulating CD200 and a lower expression of the CD200 mRNA in the secretory phase in the endometrium of the control group [69]. Abramiuk et al. found higher percentages of CD4^+^, CD8^+^ T cells, and CD19^+^ B cells with CD200 expression in EMS patients when compared to the control group. In contrast, the EMS group represents lower percentages of lymphocyte subpopulations with CD200R expression in comparison to control individuals. They also found that the concentration of sCD200 in EMS patients was significantly lower than in the control group [70]. In the same year, Weng et al., using Western blot analysis, detected an increased expression of CD200 in ectopic tissues in patients with EMS. Using the immunohistochemistry method, they showed that CD200 was upregulated and located in endometrial stromal and epithelium cells in the ectopic EMS tissues. Moreover, researchers detected a higher level soluble form of CD200 in the serum of patients with EMS in comparison to the control individuals. Interestingly, they observed that the presence of oestrogen leads to the upregulation of the expression of CD200 in human endometrial stromal cells [66]. Synthesising the presented data, CD200/CD200R may have a role in the induction of immunosuppression in these patients.

**Figure 1:**
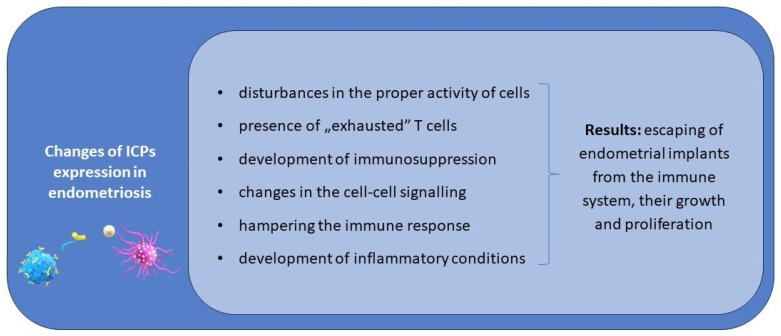
The possible role of ICPs in the pathogenesis and progression of endometriosis.

Considering the available data concerning different ICPs, it should be stressed that the knowledge concerning the signature of different ICPs is insufficient, and even the expression of the presented ICPs has not been examined on all subtypes of immune cells yet. The network of signalling by ICPs between cells and the interrelationship of modulation is complex, depending not only on inhibitory but also co-stimulatory ICPs, and there are no available reports assessing the expression of co-stimulatory ICPs and their ligands on immune system cells in endometriosis patients. The possible role of ICPs in the pathogenesis of endometriosis is presented in Figure 1.

## 9. The Possible Implication between ICPs and Microbiota in Patients with Endometriosis—It Is a More Complicated Problem than We Have Ever Thought

Until now, we underestimated the role of the microbiome in EMS pathogenesis. The microbiome consists of genomes of bacteria, archaea, eukaryotes, viruses, and fungi. Maintaining a healthy balanced microbiome is important in the homeostasis of the body and is critical for nutrient absorption. Moreover, it regulates the epithelial barrier integrity of the gut and the proper response of the immune system. Now, emerging data indicate that changes in the composition and function of the microbiome may lead to dysbiosis, which is linked to different chronic conditions, metabolic or inflammatory diseases, and cancer [71].

Recent evidence suggests that the microbiome may also be implicated in the pathogenesis of EMS. However, based on the available literature data, it is difficult to recognise if the presence of microbiome dysbiosis in patients with endometriosis is a cause or a consequence of the disease. On the other hand, changes in the microbiome can be an important factor during EMS development through enhancing systemic and local inflammation, modulating the immune response and changes in metabolism, impacting the level of circulating hormones like oestrogen through estrobolome, altering the apoptosis of cells, having involvement in oxidative stress, and increasing angiogenesis [72].

A special role is assigned to estrobolome—a collection of genes encoding oestrogen-metabolizing enzymes (β-glucosidases and β-glucuronidases), responsible for the metabolism and bioavailability of oestrogen. The enzymes mentioned below, via deconjugating oestrogen, lead to its reabsorption through enterohepatic circulation. It may result in enhancing the circulating levels of oestrogen and the presence of the hyperoestrogenic state in patients with EMS, promoting the development/progression of the disease [73]. It is widely known that oestrogen plays a crucial role in both the development and progression of EMS, and it is often called an oestrogen-dependent disease [74].

One of the most surprising discoveries was the observation that the microbiome can be implicated in the oncogenesis process and the immune response, hampering malignant transformation. It can influence immune checkpoint inhibitors (ICI) treatment, radiotherapy, and chemotherapy efficacy, and can also have impacts after the surgery [75]. Emerging data have shown that not only dysbiosis but also changes in microbial diversity are responsible for a poorer response during ICI treatment and survival outcomes in cancer patients. The microbiome ecosystem can modulate the response to ICI therapy through hampering inflammatory cytokines, reducing the percentage of Tregs, stimulating the maturation of DCs and the infiltration of T cells into the tumour microenvironment (TME), and impacting major histocompatibility complex class I/II (MHC I/II) genes. Recent data have shown that the presence of *Lactobacillus*, *Faecalibacterium*, and *Bifidobacterium* are associated with better responses, whereas the presence of *Bacteroides* reduces the effectiveness of ICI [76]. Bacteria engaged in the development of chronic infections also have the ability to induce the expression of PD-L1 on cancer cells, taking an active part in the regulation of the TME. In head and neck cancer cell lines, researchers found that *Fusobacterium* is responsible for increasing PD-L1 protein and mRNA expression. Additionally, an upregulated level of PD-L1 was found in prostate cancer cells during the presence of the *Porphyromonas gingivalis* infection [77]. The gut microbiota is engaged in breast cancer development and progression via regulating cytokines, oestrogens, and steroid hormones, and through controlling the level of leptin and insulin [73]. Also, microbial metabolites, which are present in the intestine, play an important role in the education of inflammatory cells and the modulation of human systemic immunity. On the one hand, they may favour cancer development or progression similarly to garlic acid, lithocholic acid, cadaverine, and, as mentioned before, deconjugated oestrogens, but, on the other hand, they can suppress cancerogenesis through affecting the response and efficacy of ICI therapy like kynurenine tridecane, lysine, inosine, or nicotinic acid [77].

Considering the influence of the gut microbiome on immunomodulation, cancer, and chronic infections, we supposed that the microbiome and the presence of dysbiosis may influence disturbances in the immune system and ICPs expression in patients with EMS, but, in the existing literature, there is no available data. This topic constitutes an extremely huge gap in the pathogenesis of this disease. Further etiological understandings of the interplay between immune cells and microorganisms and a possible role of the microbiome would provide the groundwork to identify more effective methods which will be crucial in developing novel strategies in endometriosis treatment.

## 10. The Future Clinical Perspectives of ICPs in Endometriosis

It is estimated that EMS affects about 170 million women. The higher prevalence of this disease was observed in women suffering from subfertility, dysmenorrhea, and pelvic pain. Low overall wellbeing, poor sleep quality, low productivity at work, dysfunction in daily activities, and episodes of depression, anxiety, and partner relationship problems are all important consequences of the presence of endometriosis. The economic costs of this disease are extremely huge, like the diagnostic process, surgery, hospitalization, monitoring tests, physician visits, and medications [78].

Although EMS is a common disease, its pathogenesis is unsolved. Implants are endometrial-like tissue outside of its normal location, but they can behave like cancer cells, sharing similarities with them such as uncontrolled growth, the ability to invade other tissues, proliferation via blood and lymphatic vessels, or decreased apoptosis, making EMS difficult to control [65]. Areas of great interest among etiopathogenetic theories are that the altered immune system activity contributes to the development of EMS, or that the aberrancies in them are a consequence of the presence of the disease [79], as well as the potential hereditary predisposition to defective immune cell activity.

**Figure 2:**
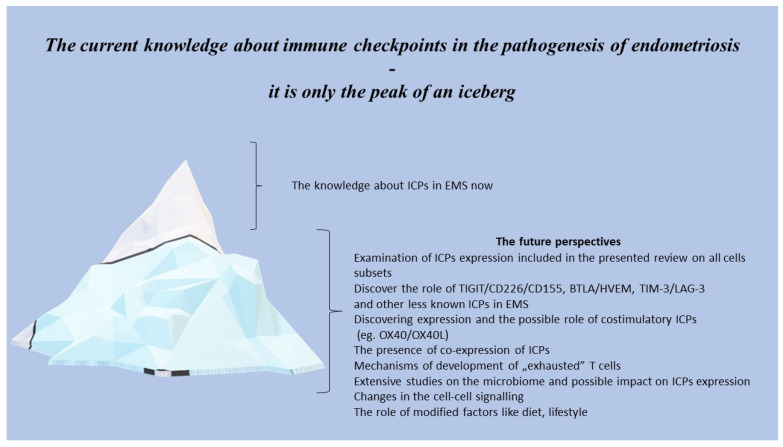
The future perspectives of ICPs in the pathogenesis of endometriosis.

The presented narrative review shows how extremely complicated and complex immune system disorders are in patients with endometriosis, including not only changes in immune cells and disturbances in the signalling provided by ICPs, but also other factors which can influence the ICPs’ expression, like oestrogen and the microbiome. The current knowledge concerning dysregulating the immune system and ICPs expression in patients suffering from EMS seems to be only the tip of an enormous iceberg (Figure 2).

Performing the extensive multiparametric analysis of the expression of ICPs and functional studies on immune cells in patients with endometriosis has filled gaps in our understanding of the pathogenesis, diagnosis, treatment, and prognosis of this disease. There are potential applications in novel therapeutic strategies via blocking co-inhibitory signals and restoring the effector activity of immune cells, preventing T and NK cells exhaustion/apoptosis. In addition, the soluble form of ICPs in the peripheral blood like Gal-9 can be implemented and used as an easy-to-obtain helpful diagnostic marker.

We must be aware that EMS should be treated with a multidisciplinary vision that includes pharmacological treatment, diet, and psychological support. The implementation of ICP-based therapy may revolutionise the treatment through recovering the proper activity of effector immune cells, supporting the antigen-presenting process as a result preventing the recurrence of the disease. It significantly improves the quality of women’s lives. Moreover, a higher percentage of successfully cured women will greatly reduce the costs of healthcare and hospitalization, elevating the overall wellbeing.

## 11. Conclusions

In the current review, we discussed key findings from the emerging literature, describing associations between ICPs and their possible implication in the pathogenesis of endometriosis. EMS is still an enigmatic and highly multifactorial disease affecting young women, involving immunological backgrounds mediated by interactions within the family of the immune checkpoint. To date, numerous studies to elucidate the exact mechanism of endometriosis development have been performed worldwide. However, it remains unclear why this disease develops in only 10% of women in their reproductive age. One of the fundamental problems with endometriosis is the limited efficacy of the current treatment options, which focus more on treating the symptoms rather than the causes of the disease. Both the upregulation and downregulation of the expression of ICP receptors and their ligands on immune system cells may lead to serious disturbances in the immune response and homeostasis in patients with EMS.

The elevated expression of ICPs, especially on peritoneal cells, may be associated with the reduced effector function of T cells, leading to their anergy, apoptosis, or ‘exhaustion’, and the impaired antigen-presenting capacity, resulting in the ineffective removal of endometrial implants from the peritoneal cavity in patients suffering from EMS.

Considering the available studies and knowledge regarding ICPs’ ability to regulate the activity of immune cells and the modulation of the adaptive and innate immune response in patients with EMS, we conclude that further extensive studies are needed to investigate the possible clinical application of ICPs in EMS treatment strategies. Immunotherapy based on ICPs will open new doors, especially in terms of the treatment of recurrent cases of the disease.
